# Case Report: Evolution of *KIT* D816V-Positive Systemic Mastocytosis to Myeloid Neoplasm With *PDGFRA* Rearrangement Responsive to Imatinib

**DOI:** 10.3389/fonc.2021.734025

**Published:** 2021-11-30

**Authors:** Mariarita Sciumè, Giusy Ceparano, Cristina Eller-Vainicher, Sonia Fabris, Silvia Lonati, Giorgio Alberto Croci, Luca Baldini, Federica Irene Grifoni

**Affiliations:** ^1^ Hematology Unit, Fondazione Istituto di Ricovero e Cura a Carattere Scientifico (IRCCS) Ca’ Granda Ospedale Maggiore Policlinico, Milan, Italy; ^2^ Postgraduate Medical School of Hematology, Università degli Studi di Milano, Milan, Italy; ^3^ Endocrinology and Diabetology Units, Department of Medical Sciences and Community, Fondazione Istituto di Ricovero e Cura a Carattere Scientifico (IRCCS) Ca’ Granda Ospedale Maggiore Policlinico, Milan, Italy; ^4^ Division of Pathology, Department of Pathophysiology and Transplantation, Fondazione Istituto di Ricovero e Cura a Carattere Scientifico (IRCCS) Ca’ Granda Ospedale Maggiore Policlinico, Milan, Italy

**Keywords:** systemic mastocytosis, myeloid neoplasm with *PDGFRA* rearrangement, imatinib, *KIT* D816V mutation, clonal evolution

## Abstract

Systemic mastocytosis (SM) is a rare neoplasm resulting from extracutaneous infiltration of clonal mast cells (MC). The clinical features of SM are very heterogenous and treatment should be highly individualized. Up to 40% of all SM cases can be associated with another hematological neoplasm, most frequently myeloproliferative neoplasms. Here, we present a patient with indolent SM who subsequently developed a myeloid neoplasm with *PDGFRA* rearrangement with complete response to low-dose imatinib. The 63-year-old patient presented with eosinophilia and elevated serum tryptase level. Bone marrow analysis revealed aberrant MCs in aggregates co-expressing CD2/CD25 and *KIT* D816V mutation (0.01%), and the *FIP1L1-PDGFRA* fusion gene was not identified. In the absence of ‘B’ and ‘C’ findings, we diagnosed an indolent form of SM. For 2 years after the diagnosis, the absolute eosinophil count progressively increased. Bone marrow evaluation showed myeloid hyperplasia and the *FIP1L1-PDGFRA* fusion gene was detected. Thus, the diagnosis of myeloid neoplasm with *PDGFRA* rearrangement was established. The patient was treated with imatinib 100 mg daily and rapidly obtained a complete molecular remission. The clinical, biological, and therapeutic aspects of SM might be challenging, especially when another associated hematological disease is diagnosed. Little is known about the underlying molecular and immunological mechanisms that can promote one entity prevailing over the other one. Currently, the preferred concept of SM pathogenesis is a multimutated neoplasm in which *KIT* mutations represent a “phenotype modifier” toward SM. Our patient showed an evolution from *KIT* mutated indolent SM to a myeloid neoplasm with *PDGFRA* rearrangement; when the eosinophilic component expanded, a regression of the MC counterpart was observed. In conclusion, extensive clinical monitoring associated with molecular testing is essential to better define these rare diseases and consequently their prognosis and treatment.

## Introduction

Systemic mastocytosis (SM) is a heterogeneous group of neoplasms characterized by abnormal expansion of clonal mast cells (MCs) in the bone marrow (BM) and other extracutaneous organ-systems (1).

According to the World Health Organization (WHO) classification, the diagnosis of SM is established in presence of the major criterion and one minor criterion or at least three minor criteria. The major criterion is fulfilled by the detection of multifocal clusters of MCs (aggregates ≥15) in one or more extracutaneous organs (usually BM), while the minor criteria include aberrant MC expression of CD25 and/or CD2, abnormal morphology of MCs, KIT mutation D816V, and a persistent serum tryptase level ≥20 ng/ml (1, 2).

There are five subtypes of SM: indolent SM (ISM), smoldering SM (SSM), SM with an associated hematological (non-MC lineage) neoplasm (SM-AHN), aggressive SM (ASM), and mast cell leukemia (MCL) (1).

The diagnosis of ISM can be established if <2 B findings and no C findings are detected; SSM is defined by ≥2 ‘B’ findings and no ‘C’ findings. ASM is characterized by one or more C findings, while MCL is defined by MCs ≥20% on marrow smears (1, 2).

There are three types of ‘B’ findings: MC infiltration >30% on bone marrow biopsy and serum total tryptase >200 ng/mL; hepatomegaly with normal liver function, palpable splenomegaly without hypersplenism, and/or lymphadenopathy; signs of dysplasia or myeloproliferation in non-MC lineage. The six ‘C’ findings are cytopenias; hepatomegaly with impairment of liver function, ascites, and/or portal hypertension; palpable splenomegaly with hypersplenism; malabsorption with weight loss due to gastrointestinal MC infiltrates; large osteolytic lesions (1, 2).

The goals of ISM treatment are symptom control, severe anaphylaxis prophylaxis, and osteoporosis treatment, while the advanced forms may require cytoreductive therapy. Historically, cytoreductive agents include interferon-α and cladribine. Allogeneic stem cell transplant could be considered in SM-AHN when the associated hematologic neoplasm has an indication of transplantation and in relapsed/refractory ASM or acute MCL (2). With the advent of the tyrosine kinase inhibitors, many efforts have been made to find a proper inhibitor of SM *KIT*–driver mutation. Imatinib still plays a role in the treatment of rare SM cases that are *KIT* D816V-unmutated, while more recently midostaurin has been shown to induce major clinical responses in advanced SM regardless of *KIT* mutational status (2, 3).

**Figure 1:**
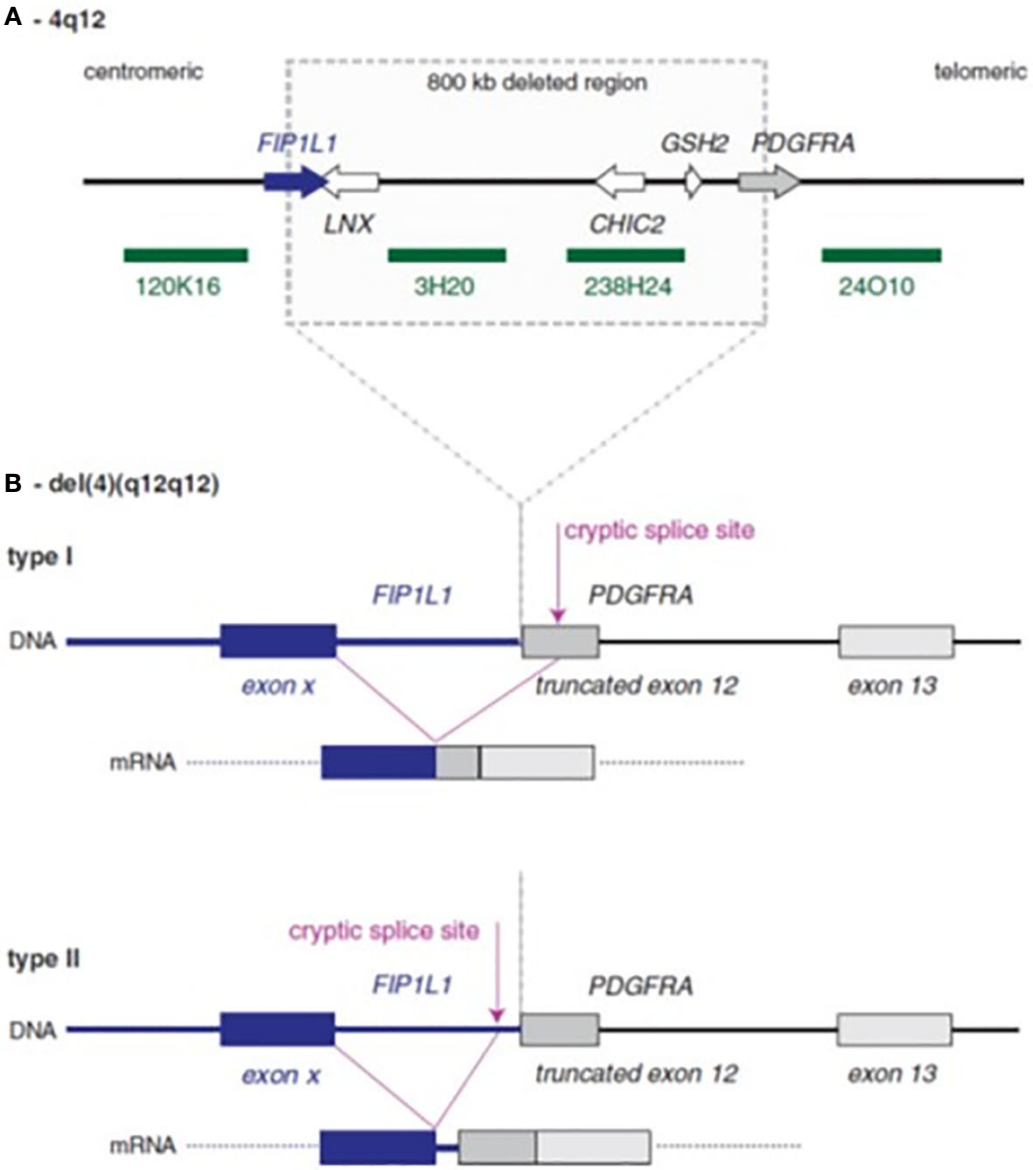
Schematic representation of *FIP1L1–PDGFRA* rearrangement. **(A)** Normal 4q12 region with sites of deletion in cases with the *FIP1L1–PDGFRA* fusion. The four green bars denote probes that can be used to detect the deletion by fluorescence *in situ* hybridization. **(B)** The consequences of deletion: *FIP1L1* usually breaks within an intron, while *PDGFRA* always breaks within exon 12. To obtain splicing between *FIP1L1* and *PDGFRA*, cryptic splice sites need to be used, because the normal splice site in the exon 12 is removed by the deletion. This cryptic splice site could be located either within exon 12 of *PDGFRA* (type I fusion) or within the intron of *FIP1L1* (type 2 fusion).

Among the myeloid neoplasms, the WHO classification recognizes the family of the myeloid/lymphoid neoplasms with eosinophilia and rearrangement of *PDGFRA*, *PDGFRB*, and *FGFR1*, or with *PCM1-JAK2.* These are rare diseases characterized by a fusion gene or a mutation resulting in the expression of aberrant tyrosine kinases. Eosinophilia (≥1.5x10^9^/L) is one of the most common features of these neoplasms. In the subgroup associated with *PDGFRA* rearrangement, the most common genetic abnormality is the *FIP1L1-PDGFRA* gene fusion, caused by 4q12 deletion (**Figure 1**) (1, 4–6). Patients frequently complain of fatigue, pruritus, and symptoms related to eosinophilic infiltrates in different organs; splenomegaly and hepatomegaly are common findings (4–6).

The natural history of *PDGFRA*-rearranged neoplasms has been dramatically altered by imatinib and the dosage of 100 mg daily could be sufficient to elicit a complete molecular response in most of the patients (7–9).

Herein, we report the clinical features and the management of a patient with *KIT* D816V-positive SM who subsequently developed a myeloid neoplasm with *PDGFRA* rearrangement with a complete molecular response to low-dose imatinib.

All procedures were in accordance with the ethical standards of the responsible committee on human experimentation (institutional and national) and with the Helsinki Declaration of 1975, as revised in 2013.

## Case Report

A 63-year-old Caucasian male patient was referred to hematological investigation because of eosinophilia.

His medical history was unremarkable except for post-infectious glomerulonephritis in childhood and flushing episodes that started one year before the hematological evaluation. No pathological findings were reported at physical examination. Previous blood tests revealed a gradual increase of absolute eosinophils count over two years. Recent laboratory examinations showed white blood count (WBC) 8.2x10^9^/L with absolute eosinophil count 2.2x10^9^/L (27%), serum tryptase level 26 ng/mL (normal level <5 ng/mL), normal serum lactate dehydrogenase (LDH), and normal liver and renal function.

A bone marrow biopsy detected 60% of cellularity, with myeloid hyperplasia, increased eosinophils and reticulin, and the presence of multifocal clusters of spindle-shaped MCs co-expressing CD25 and CD2 (>15 cells). Karyotype analysis was normal. Polymerase chain reaction (PCR) detected the D816V *KIT* mutation on bone marrow (variant allele frequency – VAF - 0.01%), while the *FIP1L1-PDGFRA* fusion transcript was absent.

Overall, these findings were consistent with a diagnosis of SM for the presence of the major criterion (BM MCs infiltrates) and 3 minor criteria (aberrant MCs, *KIT* D816V mutation, tryptase level >20 ng/mL). Abdominal ultrasound showed normal liver and spleen dimensions and an absence of lymphadenopathy. Dual-energy X-ray absorptiometry scan detected osteoporosis (lumbar T score -3.3, lumbar Z-score -2.6, femoral neck T-score -1.5, femoral neck Z-score -0.5).

In the absence of ‘B’ and ‘C’ findings, we concluded with an indolent form of SM. The patient did not receive specific therapy other than osteoporosis treatment.

Two years later the patient complained of a worsening of flushing and a recurrent headache. Blood examinations revealed an increase of WBC (13x10^9^/L) with an absolute eosinophil count of 5.1 x 10^9^/L. Suspecting a myeloid neoplasm with eosinophilia, a new bone marrow evaluation was performed. The histological analysis confirmed myeloid hyperplasia with a marked increase of eosinophils and 3% of MCs in rare aggregates. Aspirate smear revealed 36% of eosinophils and 6% of MCs. No aberrant MCs were detected with flow cytometry. Cytogenetic analysis showed normal male karyotype and a digital PCR was negative for *KIT* D816V mutation. Real-time PCR performed on peripheral blood detected the FIP1L1-PDGFRA fusion transcript and serum tryptase was 20 ng/ml. Thus, the final diagnosis was myeloid neoplasm with *PDGFRA* rearrangement.

Imatinib 100 mg daily was started, and after 3 months all symptoms resolved and blood tests showed WBC 5.96x10^9^/L with normal eosinophil count (0.09x10^9^/L).

BM analysis revealed the absence of eosinophils and the presence of rare MCs with normal morphology, corresponding to 4% of cellularity at the aspirate smear. Flow cytometry showed a normal MCs phenotype. *FIP1L1-PDGFRA* and *KIT* D816V mutations were negative on BM and peripheral blood.

A subsequent BM analysis, performed after 12 months of imatinib treatment, confirmed complete remission of the myeloid neoplasm, absence of aberrant MCs, and negative *FIP1L1-PDGFRA* and *KIT* D816V mutations.

**Figure 2:**
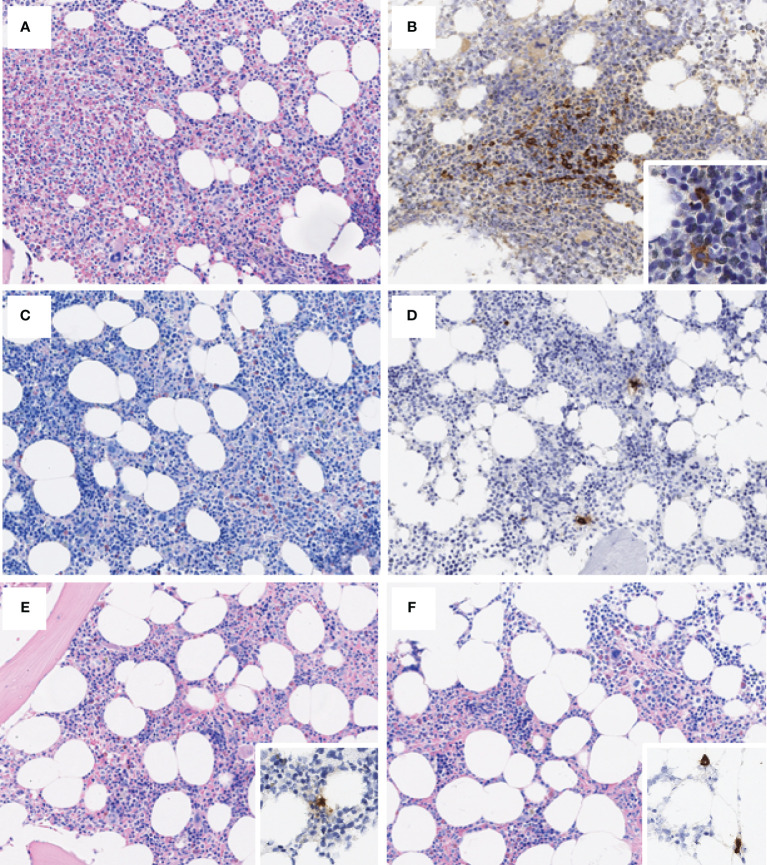
**(A)** (Giemsa, 200x) depicts a hypercellular bone marrow, featuring expansion of the eosinophilic lineage, comprising maturing to fully mature eosinophils, while tryptase stain [**(B)**; 200x] delineates the presence of scattered aggregates of epitheliod to spindled mast cells, featuring at least partial CD25-positivity (inset, 400x). Bone marrow biopsy at 3 months from Imatinib initiation [**(C)**; Giemsa, 200x] shows a reduction of cellularity, eosinophilic compartment, and mast cells [**(D)**; tryptase, 200x], which appear scattered. Restitutio ad integrum of the hematopoiesis is steadily apparent in subsequent biopsies [**(E, F)**; Giemsa, 200x], with only scattered tryptase+ cells [**(E, F)** insets].

**Table 1** and **Figure 2** report the main hematological findings at diagnosis of SM, diagnosis of myeloid neoplasm with *PDGFRA* rearrangement, and after 3, 6, and 12 months of imatinib treatment.

**Table 1:** Relevant blood and bone marrow parameters in the main time-points from diagnosis of systemic mastocytosis.

	SM Diagnosis	Myeloid neoplasm with *PDGFRA* rearrangement Diagnosis	+3 mo of Imatinib Treatment	+6 mo of Imatinib Treatment		+12 mo of Imatinib Treatment
WBC count, x10^9^/L	8.2	13.03	5.96	6.48	6.48	
Eos. in blood, x10^9^/L (%)	2.2 (27%)	5.1 (39.6%)	0.09 (1.5%)	0.15 (2.3%)	0.18 (2.8%)	
Serum tryptase, ng/mL	26	20			5	
MCs in bone marrow, histological analysis, %	CD25+ CD2+, in aggregates, not quantified	3%	2%	1%	1%	
MCs in bone marrow, aspirate smear, %	Not performed	6%	4%	0%	0%	
MCs in bone marrow, flow cytometry, %	Not performed	0.3%	2%	1%	0.2%	
Genetic markers (mononuclear cells of peripheral blood or bone marrow)	Bone marrow: *PDGFRA* - *KIT* D816V + (0.01%)	Peripheral blood: *PDGFRA* + Bone marrow: KIT D816V -	Bone marrow and peripheral blood: *PDGFRA* - KIT D816V -	Bone marrow: *PDGFRA* - *KIT* D816V -	Bone marrow: *PDGFRA* - *KIT* D816V -	

## Discussion

The clinical features of SM are very heterogenous, ranging from indolent forms to more aggressive diseases which require cytoreductive therapy (1, 2).

MCs derive from CD34+/CD117+ pluripotent hematopoietic progenitor cells in BM. They are normal residents in mucosal tissues and skin with a key role in IgE-associated disorders and acquired or innate immunity (10). More than 90% of SM patients carried a somatic mutation in different regions of *KIT* which led to structural alteration of the protein with a constitutive activation of the receptor. The *KIT* D816V mutation is the most common (11). *KIT* median VAF strongly correlates with disease activity as represented by serum tryptase level, disease subtype (indolent *versus* advanced), and survival (12).

Up to 40% of all SM cases are associated with another hematological disease, which rarely can be a myeloid/lymphoid neoplasm with eosinophilia (2, 4).

Myeloid/lymphoid neoplasms with eosinophilia and rearrangements of *PDGFRA*, *PDGFRB*, and *FGFR1* were recognized as a standalone category in the 2008 WHO classification. Subsequently, *PCM1-JAK2* was added to this family as a new provisional entity in the 2016 WHO classification (1). Besides the rare cases of SM associated with a myeloid/lymphoid neoplasm with eosinophilia, peripheral eosinophilia may affect up to 28% of all SM patients (2). A paper from 2007 compared D816V-positive SM and *FIP1L1/PDGFRA*-positive chronic eosinophilic leukemia (13). The distinguishing features for chronic eosinophilic leukemia included the degree of eosinophilia in relation to the tryptase level, the absence of dense MC aggregates, and pulmonary and cardiac symptoms. The authors concluded that the *FIP1L1/PDGFRA* gene fusion and D816V-*KIT* mutation cause different clinical syndromes and a distinction is essential for therapeutic decisions (13).

Herein, we reported a case of indolent *KIT* D816V-positive SM with eosinophilia at diagnosis, which after two years showed an evolution to myeloid neoplasm with *PDGFRA* rearrangement.

At disease onset, the clinical scenario was characterized by a moderate increase of eosinophils and serum tryptase. Genetic data revealed *KIT* D816V mutation, while rearrangements for eosinophilia were not identified. SM and myeloid neoplasms with eosinophilia were considered in the differential diagnosis.

Similar to SM, myeloid neoplasms with eosinophilia and specific rearrangements can show dysplastic eosinophils and spindle-shaped MCs. The MCs can also be CD25 positive, but do not form compact aggregates, express CD2, or carry the *KIT* D816V mutation (14). Our patient displayed all these characteristics; therefore a diagnosis of SM seemed to be the most likely despite a low *KIT* VAF.

During the follow-up, the patient complained of a worsening of flushing, headache, and eosinophilia. Detection of the *FIP1L1-PDGFRA* mutation and absence of diagnostic criteria for SM led us to a diagnosis of myeloid neoplasm with *PDGFRA* rearrangement.

When the *PDGFRA* rearrangement was identified the eosinophilic component became predominant either in the peripheral blood and bone marrow; Pardanani et al. described 12 cases of SM and chronic eosinophilic leukemia with *FIP1L1-PDGFRA*. These patients with eosinophilia were more likely to be males and exhibit a “loose” pattern of MC infiltration in BM trephines; 8 of them were also screened for *KIT* D816V and all tested negative (15).

A clonal relationship between the MC and the associated hematologic non-MC component has been sought using *KIT* and other mutations as markers of clonality (13, 14). Various studies conducted in patients with concomitant diagnosis of acute myeloid leukemia and SM demonstrated evidence that neoplastic MC and myeloid leukemic blasts are likely to develop from common hematopoietic progenitors (16, 17). Subsequent studies supported the concept of advanced SM pathogenesis as a multimutated neoplasm, in which *KIT* D816V mutation represents a “phenotype modifier” of clonal hematopoietic stem cell disorders toward SM (18). These findings challenged the concept that the SM and the associated non-MC-hematological disease arise uniformly from a pre-committed neoplastic progenitor cell harboring a *KIT* mutation and suggest that this category is highly heterogeneous (18).

Our case corroborates data by Maric and Pardanani (13, 15) who considered eosinophilic disorders and systemic mastocytosis as clinically distinct entities with different therapeutic needs. On a molecular level, further studies of next-generation and single-cell sequencing may be of benefit to clarify whether or not *KIT* mutation and *PDGFRA* rearrangement should be considered as different clones.

At the time of identification of the *FIPL1-PDGFRA* transcript, our patient did not meet the diagnostic criteria for SM: on bone marrow, MCs formed rare aggregates, no cytofluorimetric abnormal markers or *KIT* D816V mutation were identified, and serum tryptase was 20 ng/ml.

According to the literature, only skin diseases in adult patients with SM could regress, in approximately 10% of the cases, while there is no evidence of spontaneous disappearance of bone marrow findings (19). In our case, the expansion of the eosinophilic component was associated with the regression of the MC counterpart, which could be masked or really disappeared during the two years after the SM diagnosis.

ISM has a nearly normal life expectancy; symptom-directed treatment should be considered in all symptomatic patients. These therapies are directed at MC degranulation symptoms, symptomatic skin disease, and osteopenia/osteoporosis. Cytoreductive therapy could be required for advanced SM; in this patient setting, novel agents with potent inhibitory activity against KIT demonstrated significant clinical benefit and reduction of bone marrow MC burden (2).

Initially, our patient received only symptomatic therapy directed towards osteoporosis treatment. When the myeloid neoplasm with *PDGFRA* rearrangement was diagnosed, low-dose imatinib was started to avoid organ damage related to eosinophilia.

The durable hematologic and molecular remissions induced by imatinib in *FIP1L1-PDGFRA*–positive myeloid neoplasms have been corroborated by many studies; generally, 100 mg daily may be sufficient to achieve and maintain a long-term molecular response (7–9).

In our case report, after 3 months from the start of imatinib treatment bone marrow evaluation and peripheral blood tests showed normal findings with complete molecular remission of the *FIP1L1-PDGFRA*–associated neoplasm. Therapy was well-tolerated, and the patient is still in complete molecular response after 18 months of therapy.

In conclusion, SM is a rare disease with an unpredictable clinical course, especially when another associated hematological disease is diagnosed. Little is known about the underlying molecular and immunological mechanisms that can promote one entity prevailing over the other one. Extensive clinical monitoring associated with molecular testing is essential to better define the disease and consequently its prognosis and treatment.

## Patient Perspective

With the diagnosis of SM, the patient seemed very concerned. He struggled to accept that only symptomatic therapy was necessary for his rare medical condition; his concern increased even more once flushing worsened, headache became recurrent, and eosinophilia led to a diagnosis of myeloid neoplasm with *PDGFRA* rearrangement. The patient’s perspective completely changed when the low dose imatinib was started; the treatment was well-tolerated and a complete molecular response with symptom remission was obtained.

## Data Availability Statement

The original contributions presented in the study are included in the article/supplementary material. Further inquiries can be directed to the corresponding author.

## Ethics Statement

The studies involving human participants were reviewed and approved by the responsible committee on human experimentation (institutional and national). The patients/participants provided their written informed consent to participate in this study. Written informed consent was obtained from the individual(s) for the publication of any potentially identifiable images or data included in this article.

## Author Contributions

MS, GC, LB, and FIG provided the patient data regarding the hematological disease. MS, GC, and FIG wrote the manuscript. CE-V provided patient data for what concerns endocrinological aspects. GAC performed the histological examinations of bone marrow, provided iconographic materials, interpreted these data, and integrated them in the manuscript. SF and SL provided patient data regarding molecular aspects. All authors critically read and approved the final manuscript.

## Conflict of Interest

The authors declare that the research was conducted in the absence of any commercial or financial relationships that could be construed as a potential conflict of interest.

## Publisher’s Note

All claims expressed in this article are solely those of the authors and do not necessarily represent those of their affiliated organizations, or those of the publisher, the editors and the reviewers. Any product that may be evaluated in this article, or claim that may be made by its manufacturer, is not guaranteed or endorsed by the publisher.

## References

[1] Arber DA Orazi A Hasserjian R Thiele J Borowitz MJ Le Beau MM. The 2016 Revision to the World Health Organization Classification of Myeloid Neoplasms and Acute Leukemia. Blood (2016) 127(20):2391–405. doi: 10.1182/blood-2016-03-643544 27069254

[2] Pardanani A. Systemic Mastocytosis in Adults: 2021 Update on Diagnosis, Risk Stratification and Management. Am J Hematol (2021) 96(4):508–25. doi: 10.1002/ajh.26118 33524167

[3] Lim KH Pardanani A Butterfielf JH Li CY Tefferi A. Cytoreductive Therapy in 108 Adults With Systemic Mastocytosis: Outcome Analysis and Response Prediction During Treatment With Interferon-Alpha, Hydroxyurea, Imatinib Mesylate or 2-Chlorodeoxyadenosine. Am J Hematol (2009) 84:790–4. doi: 10.1002/ajh.21561 19890907

[4] Brown LE Zhang D Persons DL Yacoub A Ponnala S Cui W. A 26-Year-Old Female With Systemic Mastocytosis With Associated Myeloid Neoplasm With Eosinophilia and Abnormalities of PDGFRB, T (4,5)(Q21;Q33). Case Rep Hematol (2016) 2016:4158567. doi: 10.1155/2016/4158567 27648315 PMC5014931

[5] Shomali W Gotlib J. World Health Organization-Defined Eosinophilic Disorders: 2019 Update on Diagnosis, Risk Stratification, and Management. Am J Hematol (2019) 94(10):1149–67. doi: 10.1002/ajh.25617 31423623

[6] Gotlib J Cools J. Five Years Since the Discovery of FIP1L1–PDGFRA: What We Have Learned About the Fusion and Other Molecularly Defined Eosinophilias. Leukemia (2008) 22:1999–2010. doi: 10.1038/leu.2008.287 18843283

[7] Jovanovic JV Score J Waghorn K Cilloni D Gottardi E Metzgeroth G. Low-Dose Imatinib Mesylate Leads to Rapid Induction of Major Molecular Responses and Achievement of Complete Molecular Remission in FIP1L1-PDGFRA-Positive Chronic Eosinophilic Leukemia. Blood (2007) 109:4635–40. doi: 10.1182/blood-2006-10-050054 17299092

[8] Baccarani M Cilloni D Rondoni M Ottaviani E Messa F Merante S. The Efficacy of Imatinib Mesylate in Patients With FIP1L1-PDGFRalpha-Positive Hypereosinophilic Syndrome. Results of a Multicenter Prospective Study. Haematologica (2007) 92:1173–9. doi: 10.3324/haematol.11420 17666373

[9] Metzgeroth G Schwaab J Naumann N Jawhar M Haferlach T Fabarius A. Treatment-Free Remission in FIP1L1-PDGFRA–positive Myeloid/Lymphoid Neoplasms With Eosinophilia After Imatinib Discontinuation. Blood Adv (2020) 4(3):440–3. doi: 10.1182/bloodadvances.2019001111 PMC7013256 31995156

[10] Da Silva EZ Jamur MC Oliver C. Mast Cell Function: A New Vision of an Old Cell. J Histochem Cytochem (2014) 62(10):698–738. doi: 10.1369/0022155414545334 25062998 PMC4230976

[11] Laine E Chauvot de Beauchêne I Perahia D Auclair C Tchertanov L. Mutation D816V Alters the Internal Structure and Dynamics of C-KIT Receptor Cytoplasmic Region: Implications for Dimerization and Activation Mechanisms. PloS Comput Biol (2011) 7(6):e1002068. doi: 10.1371/journal.pcbi.1002068 21698178 PMC3116893

[12] Erben P Schwaab J Metzgeroth G Horny HP Jawhar M Sotlar K. The KIT D816V Expressed Allele Burden for Diagnosis and Disease Monitoring of Systemic Mastocytosis. Ann Hematol (2014) 93(1):81–8. doi: 10.1007/s00277-013-1964-1 24281161

[13] Maric I Robyn J Metcalfe DD Fay MP Carter M Wilson T. KIT D816V-Associated Systemic Mastocytosis With Eosinophilia and FIP1L1/PDGFRA-Associated Chronic Eosinophilic Leukemia Are Distinct Entities. J Allergy Clin Immunol (2007) 120(3):680–7. doi: 10.1016/j.jaci.2007.05.024 17628645

[14] Klion AD Noel P Akin C Law MA Gilliland DG Cools J. Elevated Serum Tryptase Levels Identify a Subset of Patients With a Myeloproliferative Variant of Idiopathic Hypereosinophilic Syndrome Associated With Tissue Fibrosis, Poor Prognosis, and Imatinib Responsiveness. Blood (2003) 101(12):4660–6. doi: 10.1182/blood-2003-01-0006 12676775

[15] Pardanani A Lim KH Lasho TL Finke C McClure RF Li C. Prognostically Relevant Breakdown of 123 Patients With Systemic Mastocytosis Associated With Other Myeloid Malignancies. Blood (2009) 114(18):3769–72. doi: 10.1182/blood-2009-05-220145 19713463

[16] Fritsche-Polanz R Fritz M Huber A Sotlar K Sperr WR Mannhalter C. High Frequency of Concomitant Mastocytosis in Patients With Acute Myeloid Leukemia Exhibiting the Transforming KIT Mutation D816V. Mol Oncol (2010) 4(4):335–46. doi: 10.1016/j.molonc.2010.04.008 PMC5527910 20471335

[17] Pullarkat V Bedell V Kim Y Bhatia R Nakamura R Forman S. Neoplastic Mast Cells in Systemic Mastocytosis Associated With T(8;21) Acute Myeloid Leukemia Are Derived From the Leukemic Clone. Leuk Res (2007) 31(2):261–5. doi: 10.1016/j.leukres.2006.03.006 16876862

[18] Jawhar M Schwaab J Schnittger S Sotlar K Horny HP Metzgeroth G. Molecular Profiling of Myeloid Progenitor Cells in Multimutated Advanced Systemic Mastocytosis Identifies KIT D816V as a Distinct and Late Event. Leukemia (2015) 29(5):1115–22. doi: 10.1038/leu.2015.4 25567135

[19] Brockow K Scott LM Worobec AS Kirshenbaum A Akin C Huber MM. Regression of Urticaria Pigmentosa in Adult Patients With Systemic Mastocytosis: Correlation With Clinical Patterns of Disease. Arch Dermatol (2002) 138(6):785–90. doi: 10.1001/archderm.138.6.785 12056960

